# ACSE-RNformer: Amplitude-Calibrated Sequence Embedding and Response-Normalized Transformer for Vibration-Based Rotating Machinery Fault Diagnosis

**DOI:** 10.3390/s26165275

**Published:** 2026-08-20

**Authors:** Yan Yan, Ting Shang, Kun Zeng, Songnan Yang, Haiyan Cheng, Wei Quan

**Affiliations:** 1 School of Automation and Information Engineering, Xi’an University of Technology, Xi’an 710048, China; 2 Dongfang Boiler Co., Ltd., Deyang 618000, China

**Keywords:** rotating machinery, fault diagnosis, Transformer, amplitude-calibrated sequence embedding, response normalization

## Abstract

To address the insufficient representation of fault characteristics in rotating machinery vibration signals, the sensitivity of conventional Transformers to variations in input response amplitudes, and the limited ability of fixed sequence embedding to preserve continuous temporal information, a rotating machinery fault diagnosis method based on Amplitude-Calibrated Sequence Embedding (ACSE) and a Response-Normalized Transformer (RNformer) is proposed. First, ACSE is designed to construct local temporal feature representations through continuous convolutional mapping, while an amplitude response estimation and adaptive amplitude calibration mechanism is employed to dynamically recalibrate the response intensity at different temporal positions. Rather than simply rescaling the signal amplitude range, amplitude calibration adaptively strengthens the feature contribution of regions associated with fault-induced impacts according to the vibration response intensity, thereby highlighting fault-sensitive information while suppressing the influence of noncritical amplitude fluctuations. In this way, continuous temporal characteristics are preserved while fault-relevant information is enhanced. Second, RNformer is constructed by incorporating a response normalization mechanism into the Transformer encoder to mitigate the interference of abnormal amplitude responses with global feature modeling, thereby improving the stability and robustness of feature representations under complex operating conditions. Finally, a lightweight channel attention mechanism is introduced to further enhance critical fault features and perform fault classification. Experiments were conducted on the Paderborn University bearing dataset and the University of Connecticut gear dataset. The proposed method achieved average diagnostic accuracies of 99.36% and 99.28%, respectively, outperforming the best-performing baseline methods by 1.82 and 1.56 percentage points. These results demonstrated the effectiveness of the proposed method for fault diagnosis.

## 1. Introduction

Rotating machinery serves as a core power component in modern industrial equipment and is widely used in aerospace, wind power generation, metallurgical manufacturing, intelligent production, and other critical fields. Its operating condition is directly related to the safety, reliability, and production efficiency of industrial systems [1]. Because rotating machinery usually operates under high-speed rotation, alternating loads, and complex working conditions, key transmission components, such as bearings and gears, are prone to wear, cracks, pitting, and spalling. If these faults are not detected and addressed in a timely manner, equipment performance may deteriorate, unplanned downtime may occur, and severe safety accidents and economic losses may be caused [2,3].

In recent years, the development of deep learning has significantly advanced fault diagnosis techniques for rotating machinery. Existing studies have primarily focused on local feature modeling. Owing to local receptive fields, weight sharing, and hierarchical feature extraction, Convolutional Neural Networks (CNNs) can automatically learn fault-related features from raw vibration signals, time–frequency images, or multi-channel sensor data, and have achieved promising performance in fault diagnosis tasks for rotating components such as bearings and gears [4,5,6]. On this basis, ResNet, ConvNeXt, Vision Transformer, and related models have further enhanced deep feature representation and have shown favorable adaptability under complex noise, limited-sample, and cross-condition diagnosis scenarios [7,8]. However, most of these models are still dominated by local neighborhood modeling. Their feature extraction capability is strongly affected by convolution kernel size, network depth, and feature aggregation strategy. As a result, their ability to model long-range dependencies across temporal scales and global dynamic patterns during fault evolution remains relatively limited [9].

To overcome the limitations of local modeling mechanisms, Transformer architectures have gradually been introduced into rotating machinery fault diagnosis [10,11,12]. Based on the multi-head self-attention mechanism, Transformers can directly establish dependencies between arbitrary positions within a sequence, thereby enabling collaborative modeling of local responses and global contextual information [13]. Jin et al. [9] applied a time-series Transformer to rotating machinery fault diagnosis and verified the effectiveness of self-attention in long-range dependency modeling. Meanwhile, long-sequence time-series models, such as Informer, Autoformer, FEDformer, and PatchTST, have improved long-sequence modeling efficiency through sparse attention, sequence decomposition, frequency-domain enhancement, and patch-based modeling, providing new perspectives for industrial time-series signal analysis [14,15,16,17]. Nevertheless, the diagnostic performance of most Transformer-based models is highly dependent on the quality of the input sequence representation. If the input tokens fail to preserve sufficient fault-sensitive information, stable and discriminative fault patterns are difficult to learn from inadequate or response-imbalanced feature representations, even when strong global modeling capability is available in subsequent layers.

Existing methods usually map raw vibration signals into token sequences suitable for Transformer processing through fixed-length patch partitioning, convolutional embedding, or multi-sensor feature fusion [18,19,20]. Although these methods have improved fault diagnosis performance, several issues remain to be further addressed. First, fixed-length patch partitioning divides continuous vibration signals into discrete segments. Although this strategy reduces the computational complexity of attention, it may weaken the continuous dynamic correlations between adjacent temporal segments, making it difficult to fully preserve continuous fault patterns. Second, convolutional embedding mainly emphasizes the extraction of local morphological patterns, while fault impulses, energy variations, and amplitude responses, which are highly discriminative for fault identification, are not sufficiently exploited. Consequently, fault-sensitive features under different response intensities are difficult to enhance adaptively. In addition, the response amplitudes of vibration signals often vary considerably across temporal positions. Local strong responses or abnormal impulses may dominate the subsequent global feature learning process, thereby affecting the ability of the Transformer to model the overall temporal structure and stable fault patterns. This reduces model robustness under complex operating conditions and noise interference. Therefore, it remains necessary to fully exploit amplitude response information while preserving continuous temporal structures and to improve the stability of Transformer input features for enhanced rotating machinery fault diagnosis.

To address these issues, this study proposes an Amplitude-Calibrated Sequence Embedding (ACSE) and Response-Normalized Transformer (RNformer) framework, namely ACSE-RNformer, for intelligent rotating machinery fault diagnosis. First, to alleviate the disruption of continuous temporal structures and the insufficient use of amplitude responses caused by fixed sequence embedding, an ACSE strategy is designed. Through continuous sequence mapping, amplitude response estimation, adaptive amplitude calibration, and sequence projection, ACSE enables collaborative modeling of continuous temporal information and amplitude response information, while enhancing fault-sensitive feature representation and effectively reducing the input sequence length. Second, to reduce the influence of input response differences on the stability of global dependency modeling, a response normalization mechanism is introduced before the Transformer encoder. The input features are corrected at the response scale, and the interference of local abnormal responses with global feature learning is reduced, thereby improving the stability and robustness of feature representation under complex operating conditions. Finally, a lightweight channel attention mechanism is incorporated to further strengthen critical fault channels and discriminative response regions, enabling accurate identification of rotating machinery fault states.

The main contributions of this study are summarized as follows:

(1) An ACSE strategy is proposed to address the difficulty of fixed sequence embedding in jointly preserving continuous temporal information and fault-sensitive responses. By integrating continuous sequence mapping, amplitude response estimation, adaptive amplitude calibration, and sequence projection, ACSE achieves collaborative modeling of local temporal structures and amplitude response information in vibration signals.

(2) An RNformer is proposed to improve the stability of global dependency modeling under input response variations. A response normalization mechanism is introduced before the Transformer encoder to correct the response scale of input features and reduce the interference caused by local strong responses or abnormal impulses during global feature learning.

(3) A unified ACSE-RNformer framework is developed for rotating machinery fault diagnosis and validated on the Paderborn University (PU) bearing dataset and the University of Connecticut (UC) gear dataset. Experimental results showed that the proposed method achieved high diagnostic accuracy on both datasets and outperformed several representative deep learning methods in classification performance.

The remainder of this paper is organized as follows. Section 2 reviews related studies on rotating machinery fault diagnosis. Section 3 describes the proposed ACSE-RNformer fault diagnosis method in detail. Section 4 presents the experimental setup, results, and analysis, and validates the effectiveness of the proposed method through comparative and ablation experiments. Section 5 concludes the paper and discusses future research directions.

## 2. Related Work

### 2.1. Signal Embedding Methods

Signal embedding is an essential step in rotating machinery fault diagnosis. Its purpose is to transform raw vibration signals into feature representations that are more suitable for model learning. Early methods mainly relied on time-domain, frequency-domain, or time–frequency-domain features, such as root mean square, kurtosis, spectral amplitude, and short-time Fourier transform, to characterize statistical variations or spectral structural changes caused by faults [21].

With the development of deep learning, convolutional embedding has become a common approach for representing one-dimensional vibration signals. Guo et al. [22] used a one-dimensional multi-channel convolutional network to directly extract local fault features from raw vibration signals. Zhai et al. [23] combined time–frequency transformation with a transfer residual convolutional network to improve fault feature representation. Zhou et al. [24] converted vibration signals into Gramian Angular Field images and integrated CNN with Vision Transformer for fault identification. These methods can effectively enhance local pattern representation, but they remain limited in preserving long-range dynamic dependencies and continuous temporal structures.

In recent years, token- or patch-based embedding methods have been introduced into Transformer-based fault diagnosis models. Nie et al. [25] proposed PatchTST, in which fixed-length patch partitioning is used to reduce the complexity of long-sequence modeling. Ko et al. [26] further adopted multi-patch representation to improve diagnostic robustness under noisy conditions. However, fixed patch embedding may split fault-related patterns, such as continuous impacts, periodic modulation, and transient responses. In addition, amplitude response variations are not sufficiently exploited, which may weaken fault-sensitive information during token mapping.

Therefore, an embedding method that can effectively enhance fault-sensitive response information while preserving continuous temporal structures is urgently needed. Such a method can provide more stable and discriminative token representations for rotating machinery fault diagnosis.

### 2.2. Transformer-Based Fault Diagnosis

Transformers establish global dependencies between different positions within a sequence through the multi-head self-attention mechanism. This design overcomes the limitation of the fixed receptive field in convolutional neural networks and provides an effective tool for modeling long-range temporal correlations in rotating machinery vibration signals [27,28]. For example, Vu et al. [29] combined a Transformer encoder with a denoising strategy to improve the stability of bearing fault classification.

Existing studies have mainly improved the applicability of Transformers to fault diagnosis from the perspectives of structural enhancement, feature fusion, and cross-condition adaptation. Li et al. [30] proposed a Transformer-based meta-learning method for bearing fault identification under limited-sample conditions. Chu et al. [31] used a Vision Transformer to fuse acoustic and vibration signals and incorporated a domain adaptation strategy to improve cross-domain diagnostic performance. These studies demonstrate the advantages of Transformers in global dependency modeling and complex fault feature extraction.

However, rotating machinery vibration signals usually contain local impacts, periodic modulation, and abrupt amplitude variations. When local strong responses or abnormal impacts exist in the input sequence, the attention distribution of a standard Transformer may be dominated by high-amplitude regions, thereby weakening the representation of other critical fault features. In addition, the diagnostic performance of a Transformer is highly dependent on the quality of the front-end token representation. If input tokens are generated by fixed patches or conventional convolutional embedding, continuous temporal structures and fault-sensitive amplitude information may not be sufficiently preserved.

## 3. Proposed Method

To fully exploit local fault patterns and global temporal dependencies in rotating machinery vibration signals, while alleviating the limitations of conventional Transformers in sequence representation quality and input response stability, an ACSE-RNformer-based fault diagnosis method is proposed in this study. The raw vibration signal is used as the input. First, continuous sequence feature extraction, amplitude response modeling, and sequence compression are performed through ACSE to construct token representations that preserve continuous temporal structures and fault-sensitive amplitude responses. Then, global temporal dependencies are modeled by RNformer, and the stability of attention learning is improved through the response normalization mechanism. Finally, the classification module integrates lightweight channel attention with a fully connected classifier to identify fault states.

### 3.1. Overall Framework

**Figure 1:**
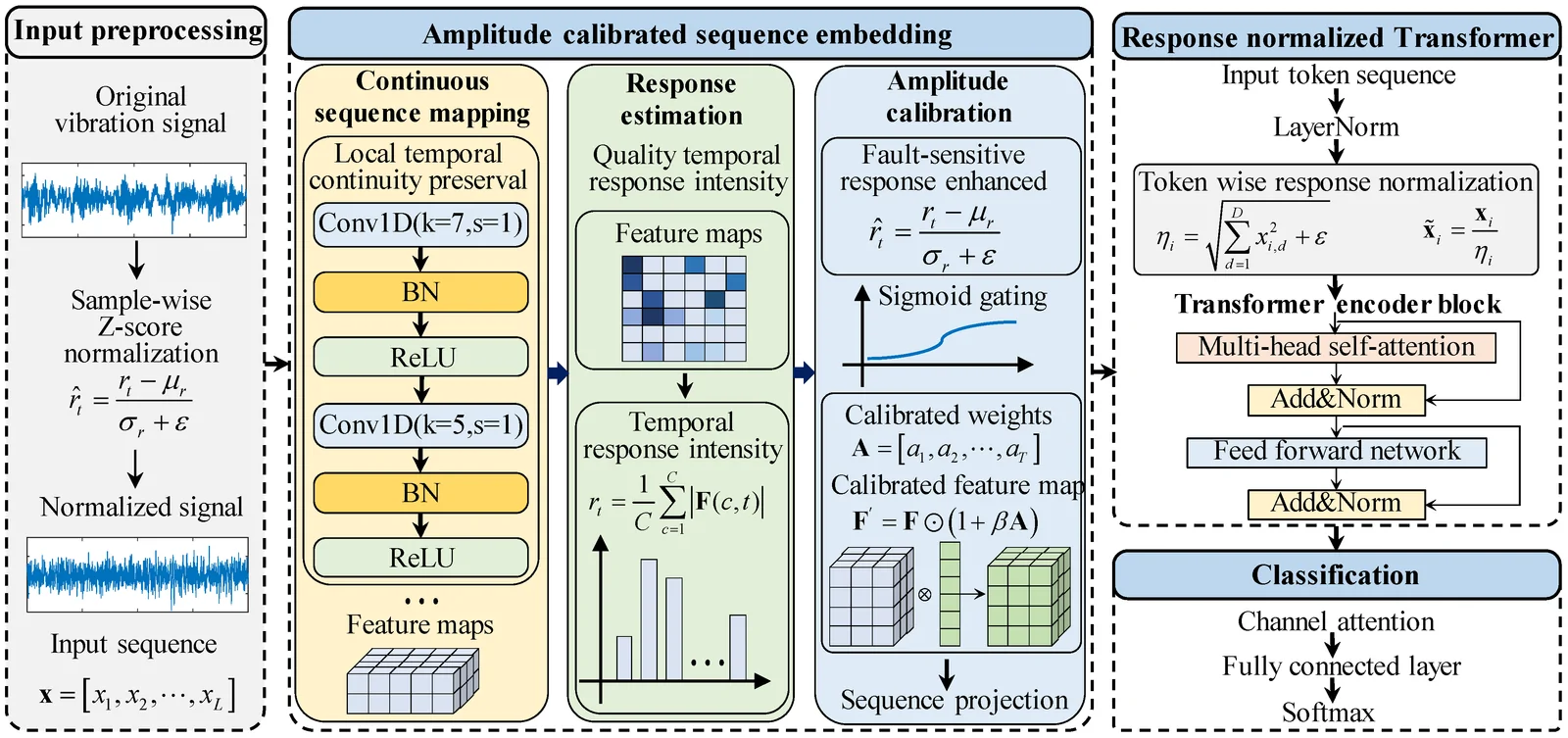
Overall framework of the proposed ACSE-RNformer for vibration-based rotating machinery fault diagnosis.

The overall framework of the proposed ACSE-RNformer is shown in Figure 1. It consists of four main stages: input preprocessing, ACSE, RNformer, and fault classification.

First, Z-score normalization is applied to the input vibration signals to reduce the influence of amplitude-scale differences among samples on model training. The normalized sequence is then fed into the ACSE module. Unlike fixed patch partitioning, ACSE extracts local temporal features through continuous convolutional mapping and enhances fault-sensitive regions by integrating amplitude response estimation with an adaptive calibration mechanism. Finally, sequence projection is used to compress the long time series into compact token representations, thereby reducing computational complexity while preserving critical temporal information.

The generated token sequence is further fed into RNformer for global feature learning. To mitigate the interference of local strong responses or abnormal impacts with the attention distribution, a response normalization mechanism is introduced before the encoder, making the response scales of different tokens more stable. On this basis, the multi-head self-attention mechanism is used to model long-range dependencies among different temporal positions, and the feed-forward network further enhances nonlinear feature representation. In this way, local response information and global contextual information are effectively integrated.

Finally, a lightweight channel attention mechanism is adopted in the classification module to adaptively enhance critical fault-related channels. Global average pooling, a fully connected layer, and a Softmax classifier are then used to output the final fault category. Overall, ACSE is designed to improve the quality of input sequence representation, whereas RNformer is designed to enhance the stability of global dependency modeling. Their collaborative design enables the proposed method to more effectively characterize fault features in complex vibration signals.

### 3.2. Amplitude-Calibrated Sequence Embedding

Transformers can effectively model long-range dependencies, but their diagnostic performance is highly dependent on the quality of the input sequence representation. For rotating machinery vibration signals, fixed-length patch embedding can reduce computational complexity, but it may disrupt the dynamic correlations among continuous impact responses. Conventional convolutional embedding can extract local patterns, but the fault-sensitive information contained in amplitude responses is not sufficiently exploited. To address these limitations, ACSE is proposed. By integrating continuous sequence mapping, amplitude response calibration, and sequence projection, ACSE enables the collaborative modeling of continuous temporal information and fault-sensitive amplitude responses. Its structure is shown in Figure 1.

The input vibration sequence is denoted as

(1) $$x=\left[x_{1},x_{2},\cdots ,x_{L}\right]\in R^{L}$$

where *L* denotes the signal length. To avoid boundary information loss caused by fixed patch partitioning, continuous one-dimensional convolutional mapping is first adopted in ACSE to construct local temporal feature representations:

(2) $$F_{1}=\delta \left(\mathrm{BN}\left({\mathrm{Conv}}_{1}(x)\right)\right)$$

(3) $$F=\delta \left(\mathrm{BN}\left({\mathrm{Conv}}_{2}(F_{1})\right)\right)$$

where Conv(⋅) denotes one-dimensional convolution, BN(⋅) denotes batch normalization, and δ(⋅) denotes the ReLU activation function. Compared with fixed patch embedding, continuous convolutional mapping gradually enlarges the receptive field while preserving local temporal continuity, thereby providing a stable feature basis for subsequent response modeling.

Faults in rotating machinery are usually accompanied by enhanced local impacts, energy concentration, and abrupt amplitude variations. Therefore, the response intensity at different temporal positions contains important discriminative information for fault diagnosis. To characterize this property, ACSE estimates the average response amplitude at each temporal position along the channel dimension:

(4) $$r_{t}=\frac{1}{C}\sum_{c=1}^{C}\left|F(c,t)\right|$$

where *C* denotes the number of channels, and F(c,t) represents the feature response of the *c*-th channel at the *t*-th temporal position. Considering that amplitude scales vary across different samples, the response sequence is further normalized as

(5) $${\hat{r}}_{t}=\frac{r_{t}-\mu_{r}}{\sigma_{r}+\varepsilon }$$

where $\mu_{r}$ and $\sigma_{r}$ denote the mean and standard deviation of the response sequence in the current sample, respectively, and ε is a small constant used to avoid division by zero. Then, amplitude calibration weights are generated through a sigmoid function:

(6) $$a_{t}=\sigma \left({\hat{r}}_{t}\right)$$

where σ(⋅) denotes the sigmoid function. The amplitude weights are then arranged as

(7) $$A=\left[a_{1},a_{2},\cdots ,a_{T}\right]$$

and are broadcast to all feature channels. Finally, the amplitude weights are applied to the continuous feature map to adaptively enhance fault-sensitive regions:

(8) $$F^{\prime }=F⊙\left(1+\beta A\right)$$

where β denotes the calibration coefficient, and ⊙ denotes element-wise multiplication. This mechanism dynamically adjusts the feature contribution at different temporal positions according to response intensity. As a result, fault-sensitive regions, such as impact responses and energy abrupt-change regions, can be more strongly represented, while noise amplification caused by indiscriminate enhancement can be avoided.

After amplitude calibration, the continuous feature map still has a relatively long temporal length. If it is directly fed into the subsequent model, the computational complexity of attention will increase rapidly. Therefore, convolutional sequence projection is further adopted in ACSE to generate compact token representations:

(9) $$Z=\mathrm{Proj}\left(F^{\prime }\right)$$

where Proj(⋅) is implemented by a one-dimensional convolution with a stride of *S* = 16. Thus, the original sequence with length *L* is finally mapped into approximately N=L/S tokens. Compared with conventional linear projection, convolutional sequence projection preserves local continuous relationships while compressing the sequence length, thereby achieving a balance between computational efficiency and feature representation capability.

Unlike conventional normalization methods, which are primarily used to adjust feature distributions, the amplitude calibration in ACSE is designed to exploit the response intensity information inherent in vibration signals to adaptively refine sequence embeddings. Rather than simply rescaling feature magnitudes, this process optimizes the representational contribution of features at different temporal positions according to fault-related response patterns, thereby enhancing fault-sensitive information.

Moreover, unlike attention mechanisms that assign weights based on feature interactions, the calibration process in ACSE is driven directly by the amplitude information of the vibration response itself and is primarily intended to improve the quality of the input sequence embeddings. Transformer attention is subsequently employed to capture global dependencies among different temporal positions.

### 3.3. Response-Normalized Transformer

Transformers rely on the multi-head self-attention mechanism to capture long-range dependencies and can effectively model global temporal correlations in vibration signals. However, rotating machinery fault signals are often characterized by local impacts, periodic modulation, and energy concentration, which may lead to significant response-amplitude differences among tokens. If attention is directly computed on such input tokens, high-response tokens may obtain excessively large correlation scores, causing the attention weights to be over-concentrated on local strong-response regions. As a result, the representation of other potentially fault-related features may be weakened. Although Layer Normalization in a standard Transformer can alleviate distribution shifts along the feature dimension, it mainly operates on the internal feature statistics of each token and is less effective in eliminating the inconsistency of overall response scales among different tokens.

To address this issue, a response-normalized Transformer, namely RNformer, is proposed. The main architecture of the Transformer encoder is retained, while a token-level response normalization mechanism is introduced before attention computation. In this way, the overall response scale of different tokens is unified, allowing attention weights to be determined more by structural correlations among tokens rather than by local amplitude magnitudes. The token sequence output by ACSE is denoted as

(10) $$Z=\left[z_{1},z_{2},\cdots ,z_{N}\right]$$

where *N* denotes the number of tokens.

First, Layer Normalization is applied to the input sequence to obtain token representations with a unified statistical distribution:

(11) $$X=\mathrm{LN}\left(Z\right)$$

where LN(⋅) denotes the Layer Normalization operation. Then, to further unify the overall response scale among different tokens, the response norm of the *i*-th token is calculated as

(12) $$\eta_{i}=\sqrt{\sum_{d=1}^{D}x_{i,d}^{2}+\varepsilon }$$

where *D* denotes the embedding dimension. Token-level response normalization is then performed as

(13) $${\tilde{x}}_{i}=\frac{x_{i}}{\eta_{i}}$$

The response-normalized input sequence is obtained as

(14) $$\tilde{X}=\left[{\tilde{x}}_{1},{\tilde{x}}_{2},\cdots ,{\tilde{x}}_{N}\right]$$

Unlike Layer Normalization, response normalization directly constrains the overall response intensity of each token. Therefore, the influence of local abnormal amplitudes on the attention distribution can be reduced, and the model is encouraged to focus more on structural dependencies among different temporal positions. Based on the normalized sequence, the query, key, and value matrices are computed as

(15) $$Q=\tilde{X}W_{Q},K=\tilde{X}W_{K},V=\tilde{X}W_{V}$$

where $W_{Q},W_{K},W_{V}$ denote the corresponding linear projection matrices. The single-head attention is calculated as

(16) $$\mathrm{Attention}(Q,K,V)=\mathrm{Softmax}\left(\frac{{\mathrm{QK}}^{T}}{\sqrt{d}}\right)V$$

where *d* denotes the dimension of each attention head. Multi-head self-attention is further adopted to learn global dependencies in different feature subspaces:

(17) $$\mathrm{MHSA}=\mathrm{Concat}(head_{1},\cdots ,head_{h})W_{O}$$

where *h* denotes the number of attention heads, and $W_{O}$ denotes the output projection matrix.

In the RNformer encoder, each encoding layer updates the features through residual connections. Let the input of the (*l* − 1)-th layer be $H^{l-1}$, where $H^{0}=Z$. The response-normalized attention update in the *l*-th layer is given by

(18) $$U^{l}=\mathrm{RN}(\mathrm{LN}(H^{l-1}))$$

(19) $${\bar{H}}^{l}=H^{l-1}+\mathrm{MHSA}(U^{l})$$

where RN(⋅) denotes the token-level response normalization operation. Then, a feed-forward network is used to further enhance nonlinear feature representation:

(20) $${\bar{H}}^{l}=H^{l-1}+\mathrm{FFN}\left(\mathrm{LN}\left({\bar{H}}^{l}\right)\right)$$

(21) $$\mathrm{FFN}(x)=W_{2}\delta \left(W_{1}x+b_{1}\right)+b_{2}$$

where $W_{1}$ and $W_{2}$ denote the two linear projection matrices, $b_{1}$ and $b_{2}$ denote the bias terms.

After multiple RNformer encoding layers, high-level feature representations containing global dependency information are obtained. Compared with the standard Transformer, RNformer unifies token response scales before attention computation, which effectively alleviates attention bias caused by local strong responses or abnormal impacts. Therefore, the stability of global dependency modeling and the representation capability of fault-sensitive features under complex operating conditions can be improved. Finally, the encoded features are fed into the channel attention module and classification module for fault category identification.

## 4. Experiments and Results

To evaluate the effectiveness of the proposed ACSE-RNformer in rotating machinery fault diagnosis, experiments were conducted on the PU bearing dataset and the UC gear dataset. The experimental datasets and preprocessing procedures are first introduced, followed by the experimental environment and training parameter settings. Then, the overall diagnostic performance of the proposed model is analyzed using confusion matrices, feature visualization, and multiple evaluation metrics. In addition, ablation experiments are performed to verify the effectiveness of ACSE and RNformer. Finally, the proposed method is compared with several representative deep learning models to demonstrate its superiority.

All experiments were conducted on a computing platform equipped with an Intel Core i9-12900K CPU (Intel Corporation, Santa Clara, CA, USA), an NVIDIA GeForce RTX 3060 GPU with 6 GB of memory (NVIDIA Corporation, Santa Clara, CA, USA), and 16 GB of RAM. The operating system was Windows 11, and all models were implemented and evaluated using MATLAB R2024b with the Deep Learning Toolbox. All comparative experiments were conducted under the same hardware and software environment to ensure a fair evaluation of computational performance.

### 4.1. Datasets

The PU bearing dataset and the UC gear dataset cover two typical types of rotating machinery components, namely rolling bearings and gears. These datasets contain different fault mechanisms and vibration response characteristics, and therefore can be used to comprehensively evaluate the generalization capability of the proposed model.

To construct unified datasets, the continuous vibration signals under each operating condition were first segmented using a fixed-length sliding window, with each segment treated as an independent sample. For the PU dataset, the window length was set to 1600 sampling points, whereas a window length of 3600 sampling points was used for the UC dataset. In both cases, the windows were arranged without overlap to ensure that adjacent samples shared no data points.

Subsequently, according to the length of the continuous vibration signals available for each operating condition, 100 independent samples were extracted from each condition for the PU dataset and 104 samples for the UC dataset, resulting in class-balanced datasets.

#### 4.1.1. Paderborn University Bearing Dataset

The PU rolling bearing dataset [32] provides high-resolution bearing vibration data. The dataset includes healthy bearings, artificially damaged bearings, and real damaged bearings generated from accelerated lifetime tests. To ensure a sufficient number of experimental samples and maintain class balance, 12 bearing conditions were selected as the research objects, including four healthy conditions, four inner-race fault conditions, and four outer-race fault conditions. The detailed fault types are listed in Table 1.

The selected PU dataset contained 1200 samples, with 100 samples for each condition. Before the experiments, Z-score normalization was applied to each sample to reduce the influence of amplitude-scale differences among samples on model training. Then, a stratified random splitting strategy was adopted to divide the samples into training, validation, and test sets at a ratio of 70%, 15%, and 15%, respectively. Specifically, 840 samples were used for training, 180 samples were used for validation, and 180 samples were used for testing. Stratified splitting ensured that the class distribution remained consistent across the training, validation, and test sets, thereby improving the reliability of model performance evaluation.

#### 4.1.2. University of Connecticut Gear Dataset

The UC gear dataset [33] contains gear vibration data collected from a two-stage gearbox test rig with replaceable gears. The sampling frequency was 20 kHz. As listed in Table 2, the dataset includes nine gear conditions: healthy, missing tooth, root crack, spalling, and five levels of chipped tooth faults. Each condition contains 104 samples, resulting in a total of 936 samples.

Before the experiments, Z-score normalization was applied to each sample to reduce the influence of amplitude-scale differences among samples on model training. Then, a stratified random splitting strategy was adopted to divide the dataset into training, validation, and test sets at approximately 70%, 15%, and 15%, respectively. Accordingly, 648 samples were used for training, 135 samples were used for validation, and 153 samples were used for testing. This splitting strategy ensured that different fault categories maintained relatively consistent class distributions across the training, validation, and test sets, thereby improving the reliability of model evaluation.

### 4.2. Experimental Settings

To ensure the fairness and reproducibility of the experimental results, all experiments were conducted using consistent data preprocessing, dataset splitting, training strategies, and evaluation metrics. Before model training, Z-score normalization was applied to each vibration sample to reduce the influence of amplitude-scale differences among samples. Table 3 summarizes the key implementation parameters and hyperparameter settings of ACSE-RNformer. A limited-range parameter search was conducted for the major hyperparameters. Specifically, the candidate kernel sizes for ACSE were {3, 5, 7}, with 5 and 7 selected for the final model; the sequence projection stride was searched over {8, 16, 32} and set to 16; the embedding dimension was searched over {32, 64, 128} and set to 64; the number of attention heads was searched over {2, 4, 8} and set to 4; the number of Transformer encoder layers was searched over {1, 2, 3} and set to 2; the feed-forward network dimension was searched over {64, 128, 256} and set to 128; and the dropout rate was searched over {0.1, 0.2, 0.3} and set to 0.2.

Accuracy, precision, recall, and F1-score were adopted to evaluate the diagnostic performance of the models. Precision, recall, and F1-score were calculated using macro averaging to comprehensively assess the recognition capability of each model across different fault categories. The corresponding formulas are given as follows:

(22) $$\mathrm{Accuracy}=\frac{\mathrm{TP}+\mathrm{TN}}{\mathrm{TP}+\mathrm{TN}+\mathrm{FP}+\mathrm{FN}}$$

(23) $$\mathrm{Precision}=\frac{\mathrm{TP}}{\mathrm{TP}+\mathrm{FP}}$$

(24) $$\mathrm{Recall}=\frac{\mathrm{TP}}{\mathrm{TP}+\mathrm{FN}}$$

(25) $$F1\text{-}\mathrm{Score}=\frac{2\times \mathrm{Precision}\times \mathrm{Recall}}{\mathrm{Precision}+\mathrm{Recall}}$$

where *TP*, *TN*, *FP*, and *FN* denote true positives, true negatives, false positives, and false negatives, respectively.

### 4.3. Experimental Results and Discussion

**Figure 2:**
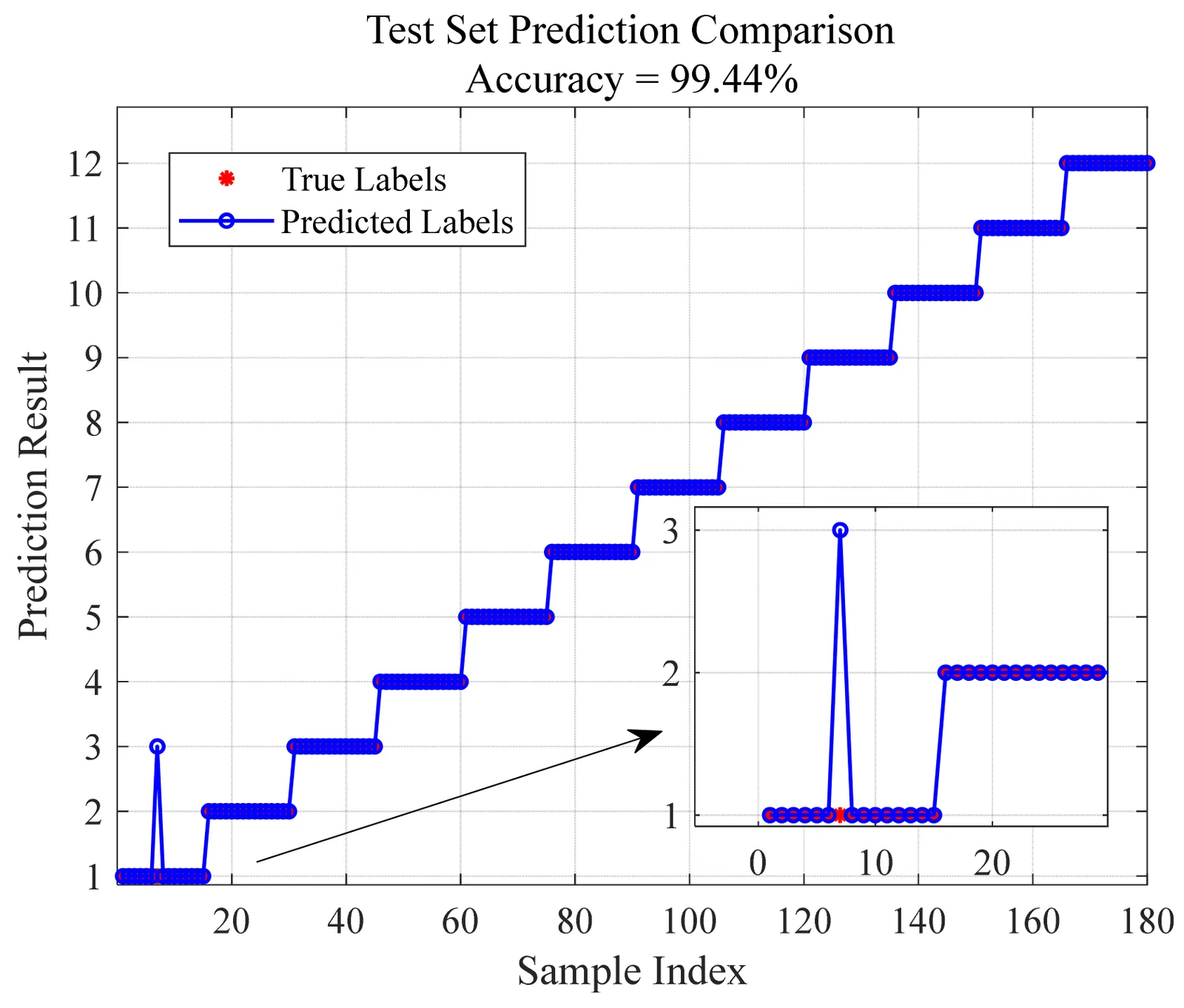
Test set prediction results on the PU dataset.

Figure 2 presents the test-set prediction results of ACSE-RNformer on the PU bearing dataset. It can be observed that the predicted labels were highly consistent with the true labels, with only one test sample being misclassified. The overall test accuracy reached 99.44%. This result indicates that the proposed method can effectively identify different bearing health conditions and fault types. This performance can mainly be attributed to two aspects. First, ACSE enhanced fault-sensitive responses in vibration signals through continuous sequence mapping and amplitude calibration. Second, RNformer reduced the interference of local abnormal amplitudes with the attention distribution through the response normalization mechanism, thereby improving the stability of global dependency modeling.

**Figure 3:**
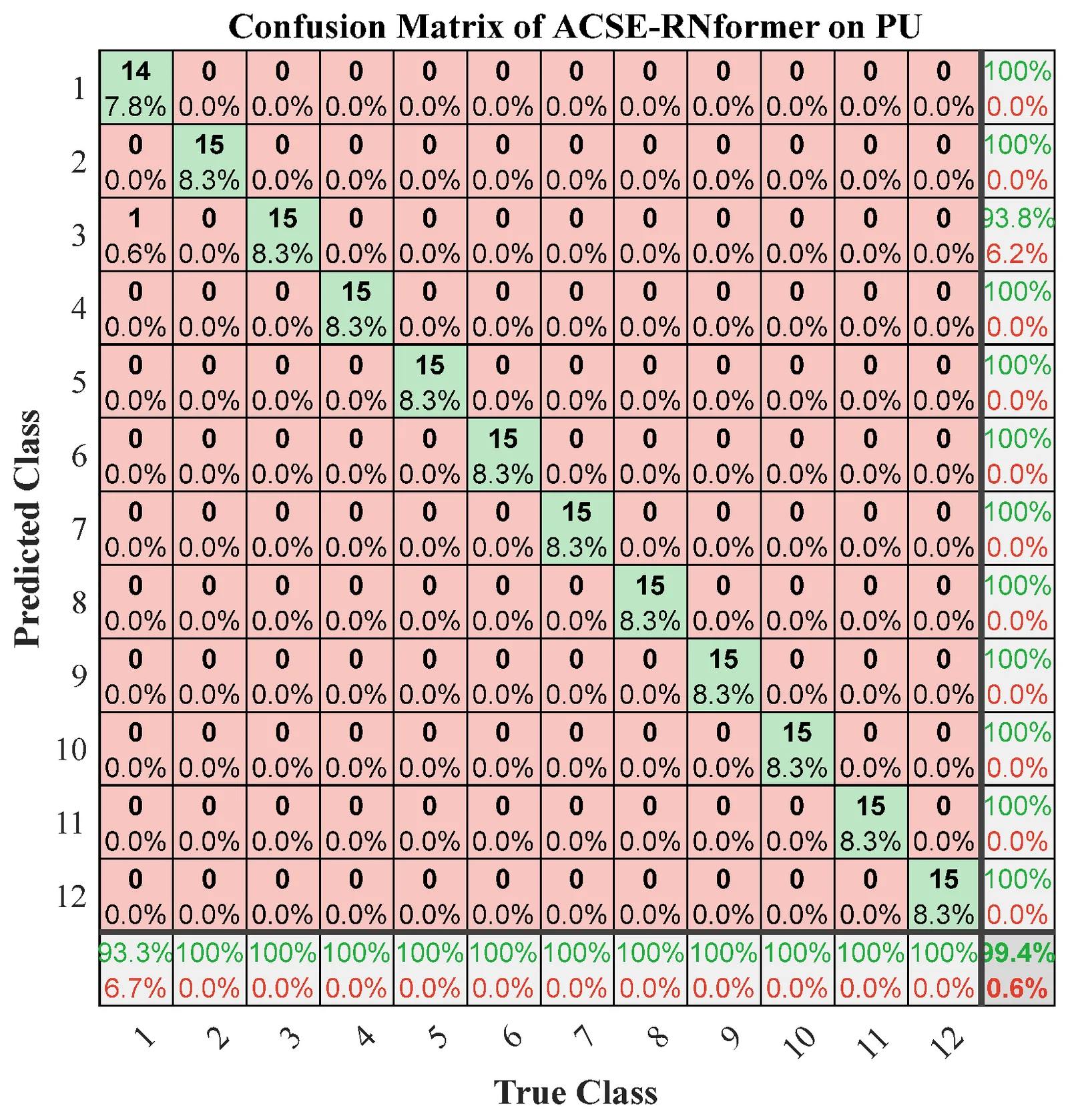
Confusion matrix on the PU dataset.

Figure 3 presents the confusion matrix of ACSE-RNformer on the PU dataset. As shown in the figure, among the 12 bearing condition classes, 11 were correctly identified without any misclassification, with only one sample being misclassified from Class 1 as Class 3. Accordingly, the Recall for Class 1 was 93.3%, while the Precision for Class 3 was 93.8%; no misclassifications occurred in the remaining classes. Further analysis indicates that a certain degree of feature overlap exists between Classes 1 and 3, possibly because the two conditions exhibit similar local impact responses, energy distributions, and time–frequency patterns, making a small number of samples difficult to completely separate in the feature space. Despite this limited confusion, the overall results demonstrate that ACSE-RNformer can effectively extract discriminative bearing fault features and maintain good separability among different fault categories.

**Figure 4:**
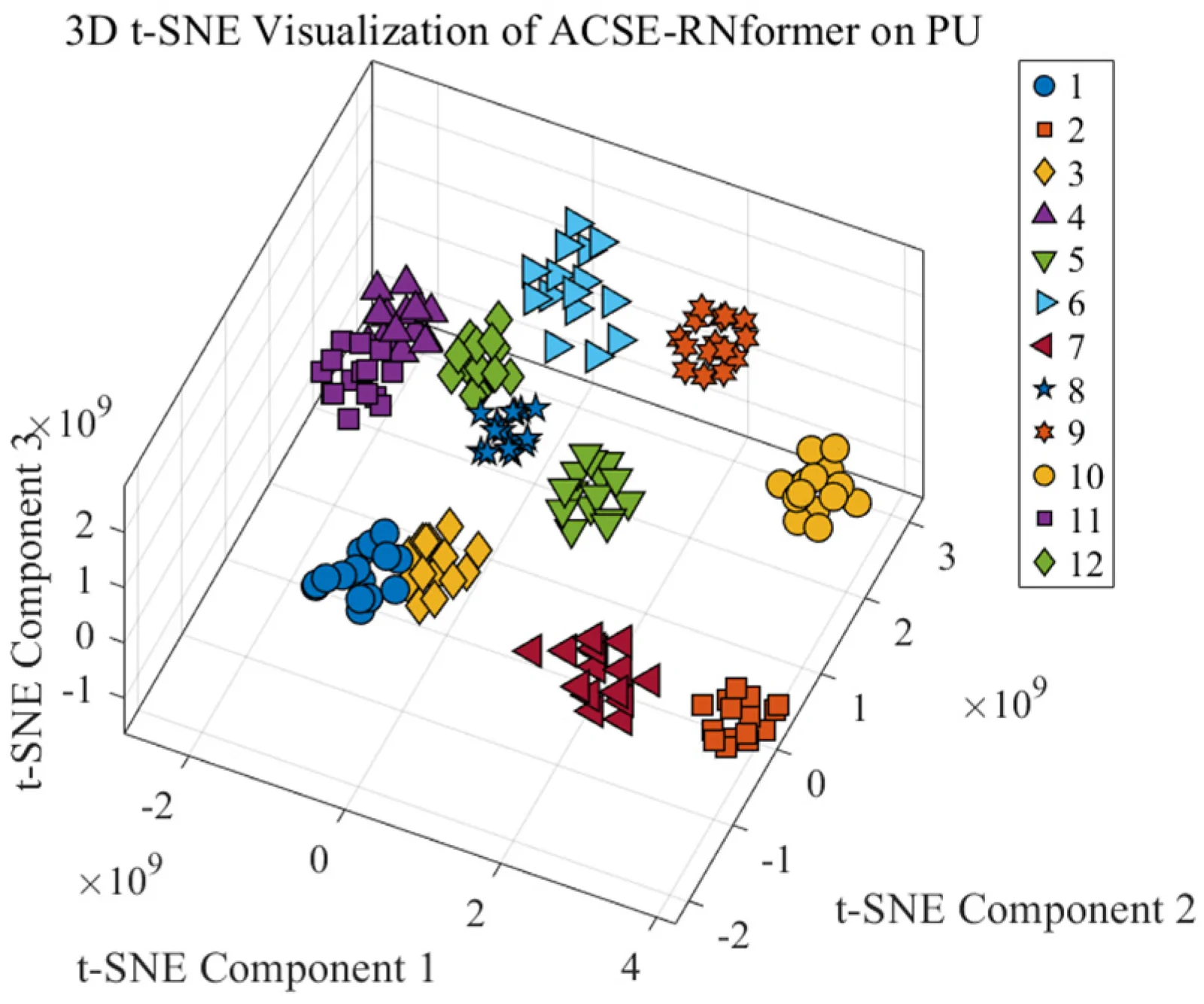
Three-dimensional t-SNE feature visualization on the PU dataset.

Figure 4 presents a three-dimensional t-SNE visualization of the features learned by ACSE-RNformer on the PU dataset. As shown in the figure, samples from the same class form relatively compact clusters in the low-dimensional embedding space, while generally clear separation boundaries are observed among different classes. The clustering regions of Classes 1 and 3 are relatively close, which is consistent with the limited misclassification observed in the confusion matrix. These results indicate that ACSE-RNformer can learn highly discriminative deep feature representations, enabling different fault conditions to exhibit well-separated distribution patterns in the low-dimensional feature space.

**Figure 5:**
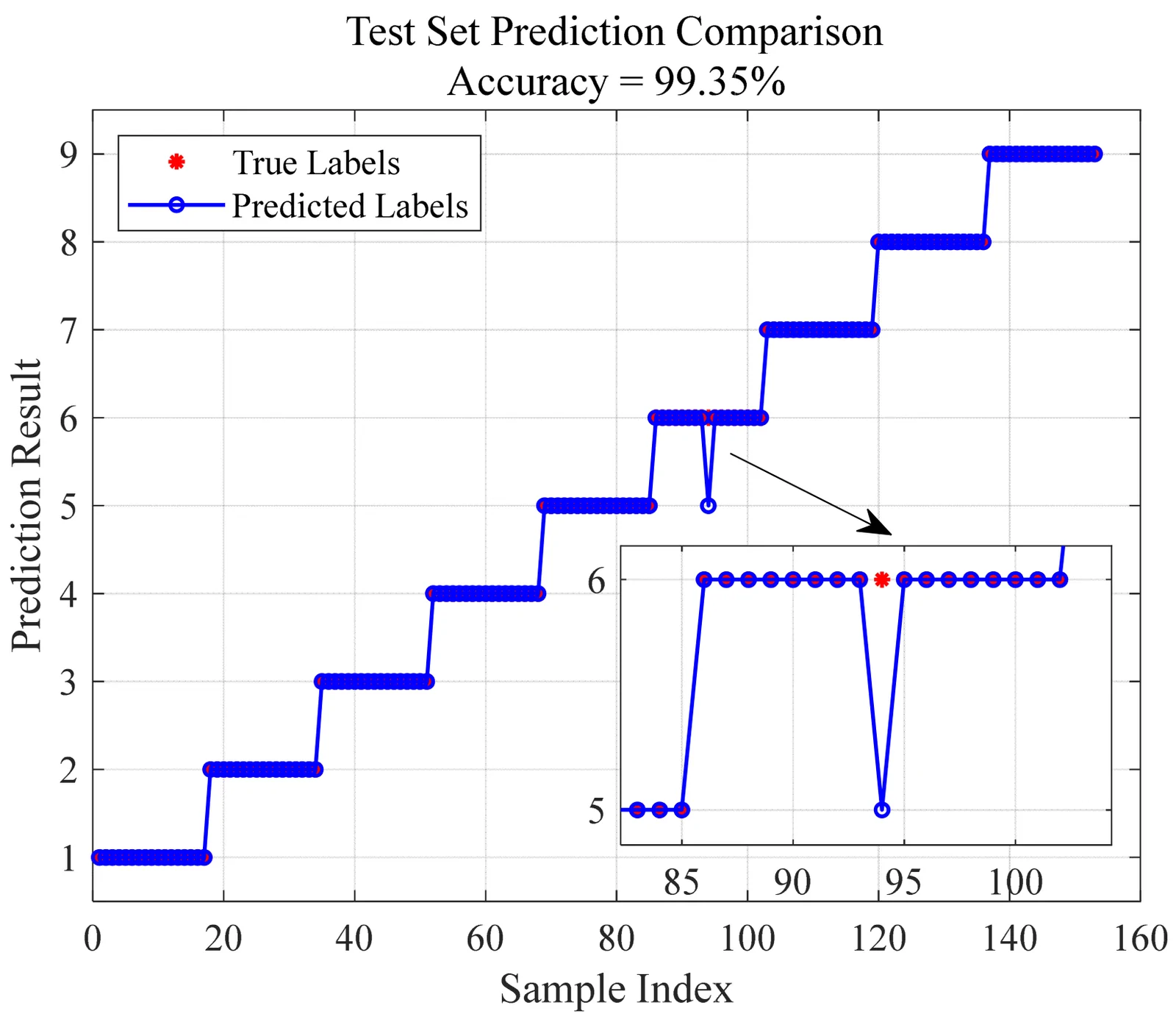
Test set prediction results on the UC dataset.

Figure 5 presents the test-set prediction results of ACSE-RNformer on the UC gear dataset. It can be observed that the predicted labels were highly consistent with the true labels, with only one test sample being misclassified. The overall test accuracy reached 99.35%. This result indicates that the proposed method can effectively identify different gear fault conditions. This performance can be attributed to the fact that ACSE enhanced fault-impact responses in gear vibration signals through amplitude-calibrated sequence embedding, whereas RNformer reduced the interference of local amplitude fluctuations with the attention distribution through the response-normalized self-attention mechanism, thereby improving the stability of global feature modeling.

**Figure 6:**
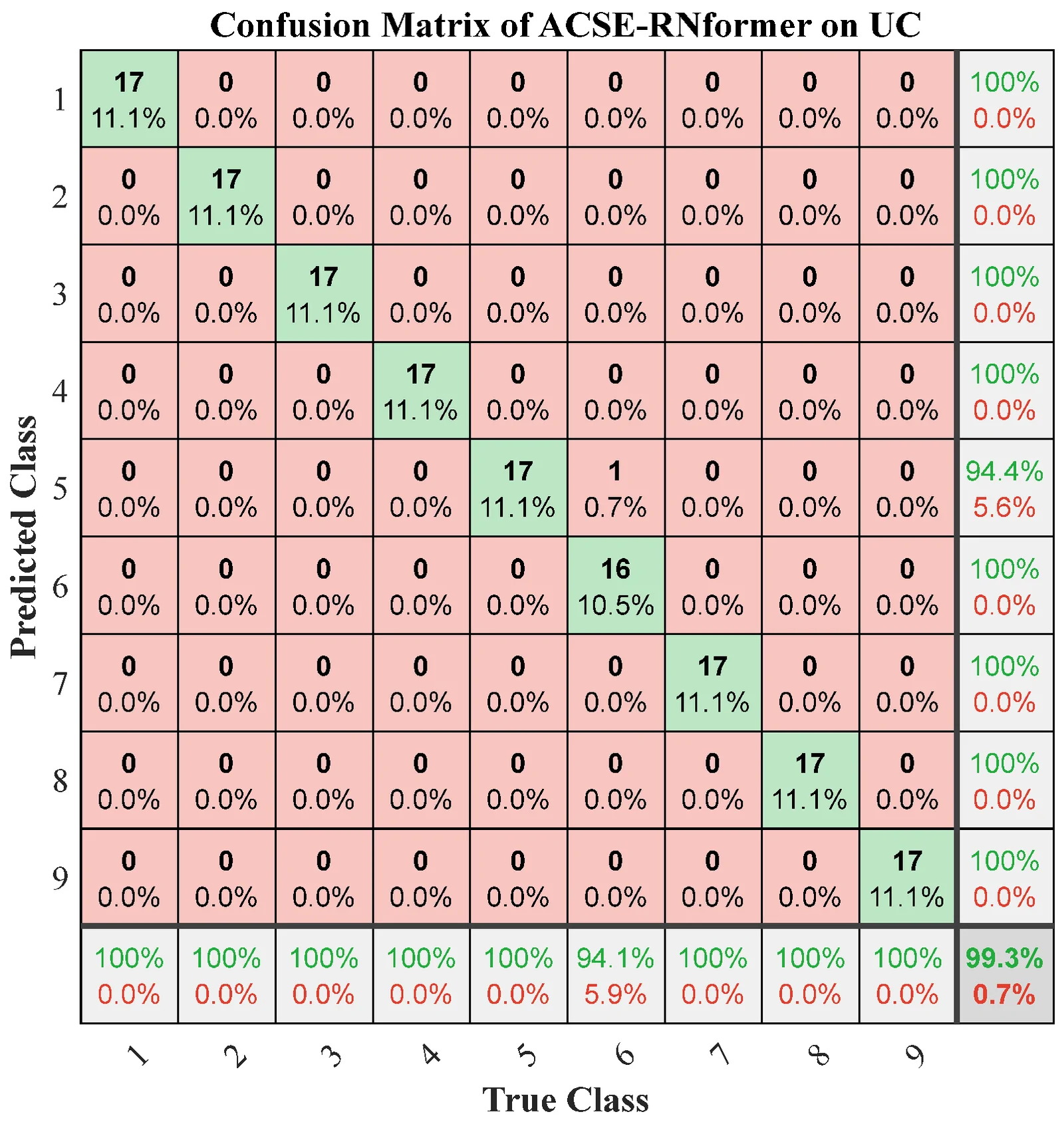
Confusion matrix on the UC dataset.

Figure 6 presents the confusion matrix of ACSE-RNformer on the UC dataset. Among the nine gear condition classes, eight were correctly identified without any misclassification, with only one sample from Class 6 being misclassified as Class 5. Accordingly, the Recall for Class 6 was 94.1%, while the Precision for Class 5 was 94.4%; no misclassifications occurred in the remaining classes. The observed confusion was concentrated between Classes 5 and 6, indicating that these two fault conditions exhibit certain similarities in their vibration response patterns, particularly in terms of local impact characteristics, amplitude variations, and spectral distributions, which increases the difficulty of distinguishing their class boundaries. Overall, ACSE-RNformer was able to learn key discriminative information from different gear fault conditions and achieve highly accurate fault classification.

**Figure 7:**
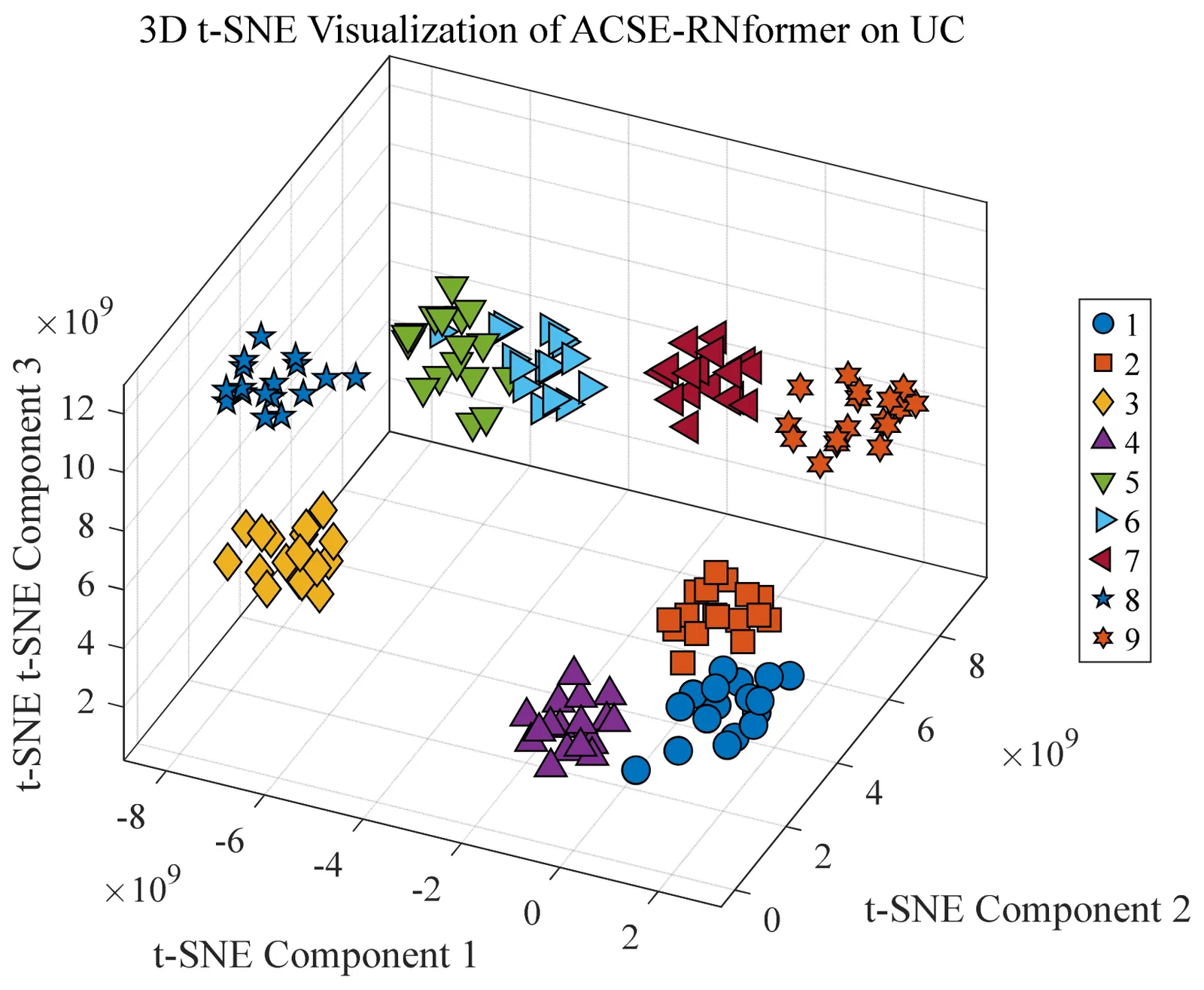
Three-dimensional t-SNE feature visualization on the UC dataset.

Figure 7 presents a three-dimensional t-SNE visualization of the features learned by ACSE-RNformer on the UC dataset. As shown in the figure, samples from the same class form relatively compact clusters in the low-dimensional embedding space, while different classes exhibit generally clear separation. In particular, the clusters corresponding to Classes 5 and 6 are relatively close, which is consistent with the single misclassification observed in the confusion matrix. This proximity may be attributed to similarities between the two fault conditions in terms of local impact responses, amplitude variation patterns, and spectral characteristics, resulting in partial overlap in the feature space. Overall, ACSE-RNformer effectively integrates amplitude response information with global temporal dependencies, enabling the learning of more stable and discriminative fault feature representations.

Overall, the experimental results on the PU bearing dataset and the UC gear dataset demonstrate that the proposed ACSE-RNformer achieved high recognition performance in two different rotating machinery fault diagnosis tasks. Only one test sample was misclassified on each dataset, and the test accuracies reached 99.44% and 99.35%, respectively. The confusion matrices show that most fault categories were completely correctly identified, with only very limited confusion between similar categories. The three-dimensional t-SNE visualizations further confirm that the learned features have clear intra-class compactness and inter-class separability. These results verify the effectiveness of ACSE and RNformer in enhancing fault-sensitive feature representation and stabilizing global dependency modeling.

For visualization, the figures presented in this section are based on a representative run among the 10 independent repetitions, whereas the quantitative results in the tables are reported as the mean ± standard deviation over all 10 runs.

### 4.4. Ablation Study

To verify the effectiveness of the two core modules, namely ACSE and RNformer, ablation experiments were conducted on the PU bearing dataset and the UC gear dataset. The results are shown in Table 4 and Table 5. The standard Transformer was used as the baseline model. ACSE, RNformer, and their combination were then introduced separately to analyze the contribution of each module to diagnostic performance. For a fair comparison, all experiments used the same data splitting strategy, training parameters, and optimization settings, and only the network structure was changed.

For the PU dataset, the standard Transformer achieved an Accuracy of 92.84% ± 0.46% and an F1-score of 92.74% ± 0.45%. After ACSE was introduced, the Accuracy and F1-score increased to 95.12% ± 0.34% and 95.05% ± 0.33%, respectively, representing improvements of 2.28 and 2.31 percentage points over the baseline. These results indicate that amplitude-calibrated sequence embedding can enhance the representation of fault-sensitive responses and improve the discriminative capability of sequence features. When only RNformer was introduced, the Accuracy and F1-score reached 97.51% ± 0.26% and 97.46% ± 0.25%, respectively, demonstrating that the response normalization mechanism can effectively mitigate the interference caused by abnormal amplitude responses and improve the stability of global feature modeling in the Transformer. When ACSE and RNformer were jointly incorporated, the model achieved an Accuracy, Precision, Recall, and F1-score of 99.36% ± 0.16%, 99.41% ± 0.15%, 99.36% ± 0.18%, and 99.37% ± 0.16%, respectively, yielding the best overall performance on the PU dataset. Compared with the standard Transformer, these four metrics were improved by 6.52, 6.23, 7.05, and 6.63 percentage points, respectively.

A consistent performance improvement was also observed on the UC dataset. The standard Transformer achieved an Accuracy of 92.43% ± 0.52% and an F1-score of 92.35% ± 0.51%. After ACSE was introduced, these values increased to 94.56% ± 0.39% and 94.49% ± 0.38%, respectively. When only RNformer was employed, the Accuracy and F1-score further increased to 97.18% ± 0.31% and 97.12% ± 0.30%. The complete model achieved an Accuracy, Precision, Recall, and F1-score of 99.28% ± 0.18%, 99.32% ± 0.16%, 99.28% ± 0.20%, and 99.29% ± 0.17%, respectively, corresponding to improvements of 6.85, 6.56, 7.32, and 6.94 percentage points over the standard Transformer. These results indicate that both ACSE and RNformer can independently improve diagnostic performance, while their joint use provides superior and more stable classification performance.

Overall, the results on both datasets demonstrate that ACSE and RNformer effectively improved diagnostic performance. ACSE mainly enhanced fault-sensitive amplitude responses in the input sequence, whereas RNformer improved the stability of global attention modeling in the Transformer. Their combination achieved the best results on both the PU and UC datasets, verifying the rationality and effectiveness of the proposed ACSE-RNformer framework.

### 4.5. Comparative Analysis

To comprehensively evaluate the effectiveness of the proposed ACSE-RNformer, seven representative fault diagnosis methods were selected as comparison models. These models represent different technical routes in the current field of intelligent fault diagnosis. The details of the comparison models are described as follows.

(1) Autoencoder (AE). AE adopted an encoder–decoder structure. The hidden-layer dimensions were set to 256, 128, and 64, and a symmetric decoder structure was used. The encoded features were fed into a fully connected layer for classification. ReLU was used as the activation function, and the dropout rate was set to 0.2.

(2) ResNet adopted a one-dimensional residual convolutional structure. The convolution kernel sizes were set to 7, 5, and 3, and the numbers of channels were set to 32, 64, and 128, respectively. Each residual block consisted of Conv1D, Batch Normalization, ReLU activation, and residual connection.

(3) Graph Convolutional Network (GCN). GCN constructed a k-nearest-neighbor graph based on sample feature similarity, where k was set to 10. Two graph convolutional layers were used, with hidden dimensions of 64 and 128, respectively. The dropout rate was set to 0.2.

(4) Kolmogorov–Arnold Network (KAN). The KAN constructed nonlinear mapping relationships based on the Kolmogorov–Arnold representation theorem. In contrast to conventional neural networks, KAN replaces fixed activation functions with learnable activation functions, thereby providing strong nonlinear function approximation capability. It has recently emerged as a representative network architecture in deep learning and has gradually been applied to fault diagnosis tasks. In this study, the hidden-layer dimensions of KAN were set to 128 and 64, the number of spline grids was set to 5, and the spline order was set to 3.

(5) iTransformer. The embedding dimension of iTransformer was set to 64, the number of attention heads was set to 4, the number of encoder layers was set to 2, and the hidden dimension of the feed-forward network was set to 128. The dropout rate was set to 0.2. Unlike the proposed method, iTransformer did not include ACSE or the response normalization mechanism.

(6) Multitask network combining CNNs and Transformers (MT-ConvFormer). MT-ConvFormer employs one-dimensional convolution to extract local fault features and a Transformer to model global dependencies. The embedding dimension was set to 64, with 4 attention heads, 2 encoder layers, and a dropout rate of 0.2, enabling joint modeling of local and global features.

(7) Multi-Patch Transformer. Multi-Patch Transformer represents vibration signals using multi-scale patches and models global dependencies among different patches through a Transformer. The embedding dimension was set to 64, with 4 attention heads, 2 encoder layers, and a dropout rate of 0.2, enhancing the representation of multi-scale local dynamic features.

To ensure a fair comparison, all comparison models were trained and tested using the same data splitting strategy, training settings, and experimental environment. The experimental results on the PU bearing dataset and the UC gear dataset are reported in Table 6 and Table 7, respectively.

For the PU dataset, the Accuracy values of AE, ResNet, GCN, KAN, and iTransformer were 82.91% ± 1.05%, 87.72% ± 0.86%, 89.94% ± 0.71%, 92.84% ± 0.58%, and 94.72% ± 0.45%, respectively, showing an overall increasing trend. MT-ConvFormer and Multi-Patch Transformer further achieved Accuracy values of 95.86% ± 0.39% and 97.54% ± 0.30%, respectively, indicating that more effective local feature extraction and temporal dependency modeling can further improve fault diagnosis performance. In comparison, the proposed ACSE-RNformer achieved an Accuracy, Precision, Recall, and F1-score of 99.36% ± 0.16%, 99.41% ± 0.15%, 99.36% ± 0.18%, and 99.37% ± 0.16%, respectively, outperforming all comparison methods. Compared with the best-performing baseline, Multi-Patch Transformer, these four metrics were improved by 1.82, 1.71, 1.98, and 1.86 percentage points, respectively, demonstrating that the proposed method can learn more discriminative fault feature representations.

A similar performance trend was observed on the UC dataset. The Accuracy values of AE, ResNet, GCN, KAN, and iTransformer were 81.46% ± 1.21%, 86.62% ± 0.94%, 89.22% ± 0.76%, 93.18% ± 0.61%, and 93.87% ± 0.50%, respectively, while MT-ConvFormer and Multi-Patch Transformer achieved 97.72% ± 0.34% and 97.21% ± 0.37%. The proposed method further achieved an Accuracy of 99.28% ± 0.18%, together with a Precision of 99.32% ± 0.16%, a Recall of 99.28% ± 0.20%, and an F1-score of 99.29% ± 0.17%. Compared with the best-performing baseline on this dataset, MT-ConvFormer, these four metrics were improved by 1.56, 1.42, 1.73, and 1.61 percentage points, respectively. These results indicate that the synergistic use of ACSE and response normalization effectively enhances fault-sensitive feature representation while improving classification performance and stability under complex vibration conditions.

Overall, the results on both datasets show that ACSE-RNformer achieved the best performance in both bearing and gear fault diagnosis tasks. This verifies its effectiveness in preserving continuous temporal structures, enhancing fault-sensitive amplitude responses, and stabilizing global dependency modeling.

To further evaluate the computational efficiency of different models, the computational costs of all models were statistically analyzed under the same hardware platform, software environment, input data, data partitioning strategy, and training settings, as shown in Table 8. Specifically, the number of model parameters, the total training time for one complete training run, and the total inference time for one complete test-set evaluation were compared. Total Training Time denotes the time required to complete a full training run, whereas Total Test-Set Inference Time denotes the time required by the trained model to complete prediction over the entire test set. Both quantities were obtained from a single complete experimental run rather than averaged over multiple runs. All models were evaluated under identical hardware, software, and testing conditions to ensure a fair comparison of computational efficiency.

As shown in Table 8, AE and ResNet have relatively low parameter counts and computational costs due to their simpler architectures. GCN and KAN incur additional computational overhead because of graph-based feature propagation and learnable function mappings, respectively. Transformer-based methods generally require more parameters and longer training times because of the attention computation. In comparison, ACSE-RNformer contains only 119.3 K parameters, which is substantially fewer than those of most Transformer-based models. This efficiency is mainly attributed to the ACSE module, which first compresses the raw vibration sequence through continuous convolutional mapping and transforms the long sequence into a compact representation, thereby reducing the computational burden of the subsequent Transformer encoding process.

Although the total test-set inference time of ACSE-RNformer is slightly higher than that of some lightweight models, it achieves superior diagnostic performance. Therefore, the proposed method maintains high fault recognition performance with controllable computational overhead, achieving a favorable balance between diagnostic accuracy and computational efficiency.

## 5. Conclusions

To address the problems that continuous temporal structures in rotating machinery vibration signals are easily disrupted, amplitude response information is insufficiently exploited, and Transformer is susceptible to local abnormal responses, an ACSE-RNformer fault diagnosis method was proposed in this study. Through the collaborative design of amplitude-calibrated sequence embedding and response-normalized Transformer, the proposed method achieved fault-sensitive feature enhancement and stable global dependency modeling.

In ACSE, continuous sequence mapping, response intensity estimation, and adaptive amplitude calibration were used to enhance fault-sensitive information, such as impact responses and energy variations, while preserving local temporal continuity. Compact token representations were then generated for subsequent global modeling. In RNformer, a response normalization mechanism was introduced before the encoder to reduce the interference of local strong responses with the attention distribution and improve the stability of global feature learning.

Experimental results on the PU bearing dataset and the UC gear dataset showed that the proposed method achieved high diagnostic accuracy under the specified test conditions and exhibited more stable classification performance than several representative deep learning models. The ablation experiments further verified the effectiveness of both ACSE and RNformer.

In summary, ACSE-RNformer provides an effective solution for intelligent rotating machinery fault diagnosis by jointly preserving continuous temporal structures, enhancing amplitude responses, and modeling global dependencies. Future work will focus on validation under cross-condition, strong-noise, and real industrial scenarios, as well as lightweight deployment and online diagnosis applications.

## Figures and Tables

**Table 1:** Fault types in the PU rolling bearing dataset.

Label	Fault Type	Fault Location	Fault Description	Number of Samples
1	K001	None	Normal	100
2	K002	None	Normal	100
3	K003	None	Normal	100
4	K004	None	Normal	100
5	KA01	Outer race	Artificial damage	100
6	KA03	Outer race	Artificial damage	100
7	KI01	Inner race	Artificial damage	100
8	KI03	Inner race	Artificial damage	100
9	KA04	Outer race	Real damage	100
10	KA15	Outer race	Real damage	100
11	KI04	Inner race	Real damage	100
12	KI14	Inner race	Real damage	100

**Table 2:** Fault types in the UC gear dataset.

Label	Fault Type	Fault Location	Number of Samples
1	Healthy	None	104
2	Missing tooth	Tooth	104
3	Root crack	Tooth root	104
4	Spalling	Gear surface	104
5	Chipping tip 5a	Tooth tip	104
6	Chipping tip 4a	Tooth tip	104
7	Chipping tip 3a	Tooth tip	104
8	Chipping tip 2a	Tooth tip	104
9	Chipping tip 1a	Tooth tip	104

**Table 3:** Key implementation details and hyperparameters of ACSE-RNformer.

Parameter	Value	Parameter	Value
PU input length	1600	Window length (UC)	3600
UC input length	3600	Window overlap	0%
Window length (PU)	1600	Sequence stride	1600/3600
Amplitude calibration coefficient	0.5	Number of attention heads	4
Numerical stability coefficient	1 × 10^−6^	Number of Transformer encoder blocks	2
Embedding dimension	64	Dropout rate	0.2
ACSE convolution kernel size	5, 7	ACSE convolution stride	1
Sequence projection kernel size	16	Sequence projection stride	16
Feed-forward network dimension	128	Optimizer	Adam
Initial learning rate	1 × 10^−3^	Mini-Batch size	64
Maximum training epochs	100	Learning rate adjustment strategy	Step decay
PU input length	1600	Window length (UC)	3600

**Table 4:** Ablation study results on the PU dataset.

Transformer	ACSE	RNformer	Accuracy (%)	Precision (%)	Recall (%)	F1-Score (%)
√			92.84 ± 0.46	93.18 ± 0.42	92.31 ± 0.52	92.74 ± 0.45
√	√		95.12 ± 0.34	95.41 ± 0.31	94.76 ± 0.39	95.05 ± 0.33
√		√	97.51 ± 0.26	97.72 ± 0.23	97.20 ± 0.30	97.46 ± 0.25
√	√	√	99.36 ± 0.16	99.41 ± 0.15	99.36 ± 0.18	99.37 ± 0.16

**Table 5:** Ablation study results on the UC dataset.

Transformer	ACSE	RNformer	Accuracy (%)	Precision (%)	Recall (%)	F1-Score (%)
√			92.43 ± 0.52	92.76 ± 0.48	91.96 ± 0.58	92.35 ± 0.51
√	√		94.56 ± 0.39	94.82 ± 0.36	94.21 ± 0.44	94.49 ± 0.38
√		√	97.18 ± 0.31	97.39 ± 0.28	96.86 ± 0.35	97.12 ± 0.30
√	√	√	99.28 ± 0.18	99.32 ± 0.16	99.28 ± 0.20	99.29 ± 0.17

**Table 6:** Evaluation metrics for various comparative networks (PU).

Model	Accuracy (%)	Precision (%)	Recall (%)	F1-Score (%)
AE	82.91 ± 1.05	83.62 ± 0.98	82.31 ± 1.12	82.79 ± 1.03
ResNet	87.72 ± 0.86	88.14 ± 0.82	87.08 ± 0.95	87.60 ± 0.87
GCN	89.94 ± 0.71	90.35 ± 0.69	89.38 ± 0.78	89.82 ± 0.72
KAN	92.84 ± 0.58	93.20 ± 0.55	92.37 ± 0.63	92.76 ± 0.57
iTransformer	94.72 ± 0.45	95.08 ± 0.42	94.31 ± 0.51	94.68 ± 0.44
MT-ConvFormer	95.86 ± 0.39	96.10 ± 0.36	95.58 ± 0.42	95.82 ± 0.38
Multi-Patch Transformer	97.54 ± 0.30	97.70 ± 0.28	97.38 ± 0.34	97.51 ± 0.29
Proposed	99.36 ± 0.16	99.41 ± 0.15	99.36 ± 0.18	99.37 ± 0.16

**Table 7:** Evaluation metrics for various comparative networks (UC).

Model	Accuracy (%)	Precision (%)	Recall (%)	F1-Score (%)
AE	81.46 ± 1.21	81.68 ± 1.16	80.52 ± 1.30	81.12 ± 1.20
ResNet	86.62 ± 0.94	87.06 ± 0.88	86.31 ± 1.05	86.70 ± 0.93
GCN	89.22 ± 0.76	89.45 ± 0.71	88.61 ± 0.84	89.04 ± 0.75
KAN	93.18 ± 0.61	93.55 ± 0.57	92.81 ± 0.67	93.10 ± 0.60
iTransformer	93.87 ± 0.50	94.61 ± 0.45	94.02 ± 0.55	94.31 ± 0.49
MT-ConvFormer	97.72 ± 0.34	97.90 ± 0.31	97.55 ± 0.37	97.68 ± 0.33
Multi-Patch Transformer	97.21 ± 0.37	97.38 ± 0.34	96.98 ± 0.41	97.16 ± 0.36
Proposed	99.28 ± 0.18	99.32 ± 0.16	99.28 ± 0.20	99.29 ± 0.17

**Table 8:** Computational efficiency comparison of different methods on the PU dataset.

Model	Total Training Time (s)	Total Test-Set Inference Time (s)	Parameter (K)
AE	9.214	0.082	86.5
ResNet	21.736	0.126	398.7
GCN	28.452	0.164	315.6
KAN	42.183	0.287	612.4
iTransformer	31.526	0.238	684.9
MT-ConvFormer	27.864	0.221	458.6
Multi-Patch Transformer	35.217	0.265	536.8
Proposed	13.538	0.201	119.3

## Data Availability

The data analyzed in this work are publicly available benchmark datasets. The Paderborn University (PU) bearing dataset [32] is available from the Bearing Data Center of Paderborn University at: https://mb.uni-paderborn.de/kat/forschung/bearing-datacenter/data-sets-and-download (accessed on 16 August 2026). The University of Connecticut (UC) gear dataset [33] is available from Figshare at: https://doi.org/10.6084/m9.figshare.6127874. The processed data and experimental results supporting the findings of this study are available from the corresponding author upon reasonable request.

## Abbreviations

The following abbreviations are used in this manuscript:

ACSE	Amplitude-Calibrated Sequence Embedding
RNformer	Response-Normalized Transformer
PU	Paderborn University
UC	University of Connecticut
CNN	Convolutional Neural Network
AE	Autoencoder
GCN	Graph Convolutional Network
KAN	Kolmogorov–Arnold Network
